# Dynamic control of eye-head gaze shifts by a spiking neural network model of the superior colliculus

**DOI:** 10.3389/fncom.2022.1040646

**Published:** 2022-11-17

**Authors:** Arezoo Alizadeh, A. John Van Opstal

**Affiliations:** Department of Biophysics, Donders Centre for Neuroscience, Radboud University, Nijmegen, Netherlands

**Keywords:** gaze saccades, motor map, midbrain superior colliculus, neural encoding, eye-head coupling, initial eye position, reference frames, non-linear pulse generator

## Abstract

**Introduction:**

To reorient gaze (the eye’s direction in space) towards a target is an overdetermined problem, as infinitely many combinations of eye- and head movements can specify the same gaze-displacement vector. Yet, behavioral measurements show that the primate gaze-control system selects a specific contribution of eye- and head movements to the saccade, which depends on the initial eye-in-head orientation. Single-unit recordings in the primate superior colliculus (SC) during head-unrestrained gaze shifts have further suggested that cells may encode the instantaneous trajectory of a desired straight gaze path in a feedforward way by the total cumulative number of spikes in the neural population, and that the instantaneous gaze kinematics are thus determined by the neural firing rates. The recordings also indicated that the latter is modulated by the initial eye position. We recently proposed a conceptual model that accounts for many of the observed properties of eye-head gaze shifts and on the potential role of the SC in gaze control.

**Methods:**

Here, we extend and test the model by incorporating a spiking neural network of the SC motor map, the output of which drives the eye-head motor control circuitry by linear cumulative summation of individual spike effects of each recruited SC neuron. We propose a simple neural mechanism on SC cells that explains the modulatory influence of feedback from an initial eye-in-head position signal on their spiking activity. The same signal also determines the onset delay of the head movement with respect to the eye. Moreover, the downstream eye- and head burst generators were taken to be linear, as our earlier work had indicated that much of the non-linear main-sequence kinematics of saccadic eye movements may be due to neural encoding at the collicular level, rather than at the brainstem.

**Results and discussion:**

We investigate how the spiking activity of the SC population drives gaze to the intended target location within a dynamic local gaze-velocity feedback circuit that yields realistic eye- and head-movement kinematics and dynamic SC gaze-movement fields.

## Introduction

### Background

A saccadic gaze shift is the rapid re-orienting movement of the eyes and head that brings the image of a peripheral visual stimulus of interest onto the fovea. A problem in the control of combined eye-head gaze shifts is that any specific displacement of gaze can be realized by infinitely many combinations of eye- and head movements. In addition, because of the different plant dynamics of the two motor systems, and because of the limited oculomotor range, not all eye-head combinations are possible or equally efficient. Typically, the amplitudes of eye- and head movements are coupled: small gaze shifts evoke small head movements, while large gaze shifts are associated with larger head movements (Guitton and Volle, 1987; Goossens and Van Opstal, 1997; Freedman and Sparks, 2000). However, the relative contributions of eyes and head to the gaze shift also depend on the initial eye orientation: if the head is initially oriented straight ahead, and the eye-in-head looks contralaterally from the target, the gaze shift will consist of a larger eye- (and hence smaller head-) movement, than when the eye looks in the ipsilateral target direction. Because the eye saccade is much faster than the head saccade, the former gaze shift will be much faster than the latter, as the eye- and head movements are not executed independently, but interact (Guitton and Volle, 1987; Goossens and Van Opstal, 1997; Freedman and Sparks, 2000; Kardamakis et al., 2010; Gandhi, 2012).

#### Kinematics

The differences in head-movement amplitude and overall gaze kinematics relate strongly to differences in the head-movement onset delay with respect to the eye movement. In the contralateral condition, the head starts later than in the ipsilateral situation and can therefore only briefly (if at all) interact with the ongoing eye movement (Volle and Guitton, 1993). However, when eye and head start nearly simultaneously, the interaction will be complete, and the slower head movement will reduce the overall gaze velocity. As a result, the well-known stereotyped main-sequence relationship, which relates the amplitude to peak velocity for eye-only saccades (Bahill et al., 1975), is no longer valid for combined eye-head gaze shifts: when a head movement accompanies the gaze shift, the gaze-peak velocity will be reduced, even though the gaze amplitude may remain the same (Guitton and Volle, 1987; Delreux et al., 1991; Goossens and Van Opstal, 1997; Freedman, 2001).

### Existing models

From the wealth of existing behavioral data on eye-head gaze shifts, obtained from human subjects (Gresty, 1974; Barnes, 1979; Zangemeister and Stark, 1982a,b; Laurutis and Robinson, 1986; Guitton and Volle, 1987; Pelisson et al., 1988; Lefèvre et al., 1992; Goossens and Van Opstal, 1997), cats (Blakemore and Donaghy, 1980; Fuller et al., 1983; Guitton et al., 1984, 1990), and monkeys (Bizzi et al., 1971, 1972; Morasso et al., 1973; Whittington et al., 1981; Tomlinson and Bahra, 1986a,b; Freedman et al., 1996), different models have been proposed, which we here briefly review.

Head-unrestrained neural recordings and microstimulation studies in cat suggested that the midbrain Superior Colliculus (SC) represents a dynamic gaze motor-error signal that acts as a common drive for the eyes and head [the so-called “common gaze-feedback” model; Guitton et al., 1990; Galiana and Guitton, 1992; reviewed by Guitton (1992)].

However, behavioral studies in humans indicated that the directions and trajectories of eye- and head movements within a gaze shift may differ substantially for large gaze shifts (Glenn and Vilis, 1992; Tweed et al., 1995) and for different relative initial eye- and head orientations (Guitton and Volle, 1987; Lefèvre et al., 1992; Goossens and Van Opstal, 1997; Freedman and Sparks, 2000). These studies therefore suggested that eyes and head are controlled by their own eye- and head motor-error signals, respectively.

This could be achieved in different ways:

The models proposed by Freedman (2001, 2008) and Kardamakis et al. (2010). Do not rely on gaze-feedback, but guarantee accurate gaze shifts by independent, yet interacting, head- and eye motor systems. However, without gaze feedback, eye- and head perturbations will affect gaze accuracy. The advantage of dynamic gaze feedback is that the system remains robust against perturbations of the eye (e.g., as a result of intervening blinks; Goossens and Van Opstal, 2000; Gandhi, 2012), or of the head (by unexpectedly applied torques; Boulanger et al., 2012). Thus, Goossens and Van Opstal (1997) proposed an alternative gaze-feedback model in which a central gaze-displacement signal was decomposed into adequate oculomotor and head-motor error signals that drive eyes and head in independent, only weakly interacting circuits.

More recently, a quite different type of model was proposed by Daye et al. (2014). In their so-called hierarchical dual-path control scheme, two parallel interacting pathways from SC and cerebellum drive the brainstem gaze- and head motor systems with a common vectorial gaze-displacement error, while the cerebellum also issues a separate head-displacement command that bypasses the SC. The brainstem short-lead burst cells in their model specify a non-linear saturating *gaze-*velocity signal, rather than *eye* velocity. As the intended movement commands for gaze and head are preprogrammed by higher (cortical) areas, the model does not incorporate the strong influence of initial eye-in-head position on the kinematics, relative timings, and metrics of eye-head gaze shifts.

This neuro-anatomically detailed model accounts for the results of head perturbations, and for the main-sequence properties observed for eye-only and eye-head gaze saccades. It can also generate (slower and longer-latency) gaze shifts without an intact SC, which was taken as a major argument to discard a pivotal role for the SC in the control of eye- and gaze saccades, as assumed in other models (Galiana and Guitton, 1992; Lefèvre et al., 1992; Goossens and Van Opstal, 1997; Freedman, 2001; Kardamakis et al., 2010; Boulanger et al., 2012). As a result, the details of the SC firing patterns do not play any significant role in the dual-path model.

However, the fact that the gaze-control system can partly recuperate, given time, from a complete and irreversible lesion of the SC does not necessarily mean that the latter is not the central controller under normal conditions and local (reversible) inactivation. Indeed, the immediate effect of a reversible bilateral SC inactivation on saccades is quite dramatic (Hepp et al., 1993), as all saccades are virtually abolished. One may instead assume that after a chronic and complete SC lesion other structures (like the frontal eye fields) could learn to take over the control (Schiller et al., 1980; Peel et al., 2020). Moreover, taking away a central role for the SC in gaze control does not readily explain the observed tight relationships between SC firing rates and instantaneous eye- and gaze-movement kinematics, described below.

### Objectives of this study

The main goals of our study are summarized as follows: (i) to account for the experimentally observed decomposition of the gaze vector into unique eye- and head movement contributions; (ii) to explain how the cumulative spike count of the SC population can drive the gaze shift in a feedforward way; (iii) to explain how the SC firing rates and eye-head control circuits determine the rich repertoire of non-linear main-sequence properties of gaze shifts, and (iv) to account for the influence of initial eye position on SC firing rates and on the gaze-shift metrics and kinematics. Instead of a preprogrammed desired head-displacement command from cortex, as in the dual-path control model (Daye et al., 2014), the head-movement contribution to the gaze shift in our model is automatically determined by the effect of eye position on the eye-head latency difference. We also suggest that both the effects of eye position on SC firing rates and on the eye- and head movement latencies may be due to a single linear modulation mechanism. As we propose a functional control model, with the aim to account for a wide variety of phenomena in the control of gaze shifts with as few assumptions as possible, we have neither attempted to exactly fit experimental data, nor to identify all its signals and transformations to particular nuclei in brainstem, cortex, spinal cord, or cerebellum.

In what follows, we first describe experimental data and the associated analysis obtained from a single-unit recording in the monkey SC during eye-head gaze shifts. Data such as these form the basis of our model.

### Superior colliculus activity during gaze shifts

**FIGURE 1:**
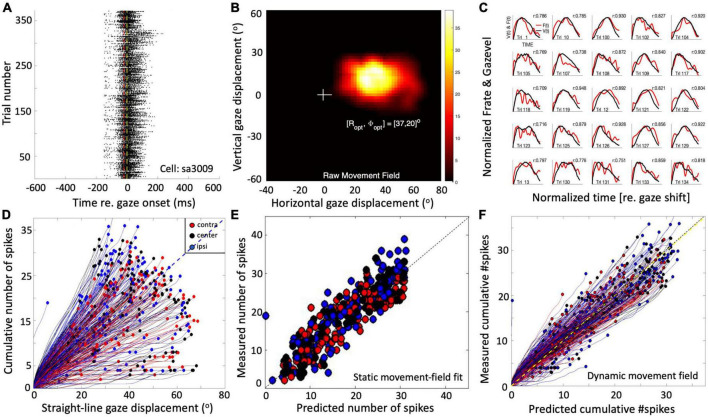
Response properties of SC neuron sa3009 for eye-head gaze shifts into and near its movement field. **(A)** Dot display of the SC bursts (371 trials) aligned with gaze-shift onset (yellow line at 0). **(B)** Raw movement field of the cell indicates that it is recruited for large rightward gaze shifts (between 20 and 70°) with a small upward component. For visual purposes, we applied a Gaussian spatial smoothing kernel around each grid point with a std of 3°. **(C)** There is a high correlation between instantaneous firing rate and gaze velocity (shifted backward by 20 ms; shown for 25 trials). For visual purposes, time axes were normalized to saccade durations and amplitudes normalized to their maximum. **(D)** The straight instantaneous gaze trajectory is linearly related to the cumulative number of spikes in the burst (thin lines). This holds for all gaze shifts (fast and slow). The slopes of the lines vary systematically with the gaze-shift vector. Colors denote the three different initial eye positions in each trial (legend). Note that ipsilateral trials (blue) tend to elicit slightly more spikes than contralateral trials (red). **(E)** Result of fitting Equation 14 (see section “Materials and methods”) to the number of spikes in the burst. Parameters: N_0_ = 31 spikes, R_*opt*_ = 36.6°, Φ_*opt*_ = 22.3°, σ_*p*_ = 0.63 mm, and ε = 0.0024 spikes/deg. Correlation fit vs. data: *r* = 0.92. The small eye-position sensitivity, ε, accounts for the eye-position effect seen in panel **(D)**. **(F)** Result of Equation 16 (see section “Materials and methods”) for the dynamic data (*r* = 0.95 for *N* = 37,679 data points).

Single-unit recordings in the midbrain SC of head-restrained (Goossens and van Opstal, 2006; Goossens and van Opstal, 2012) as well as head-unrestrained (Van Opstal and Kasap, 2019) monkeys have suggested that the population of saccade-related cells encodes a desired straight gaze trajectory by the instantaneous cumulative spike count, whereby the gaze kinematics are determined by the instantaneous neural firing rates. To illustrate this, Figure 1 exemplifies the results from a single-unit SC recording from a monkey making large head-unrestrained gaze shifts in and near the cell’s movement field (see Supplementary material, for details). Gaze saccades started from the central fixation point at straight ahead with the eye-in-head in one of three possible initial orientations: 18° left (contralateral), straight ahead, or 18° right (ipsilateral) (Supplementary Figure 1; Van Opstal and Kasap, 2019). Visual targets appeared (mostly) in the right hemifield, in and around the cell’s movement field. The top row (Figures 1A–C) provides the raw data of 371 trials. Panel 1A shows the spiking patterns for each gaze shift, aligned with gaze-saccade onset (yellow line at *t* = 0). The cell rapidly increased its firing rate around 20 ms before saccade onset (red line; the cell’s lead time). In Figure 1B, the number of spikes emitted during each gaze shift has been color-coded, clearly showing the restricted set of gaze vectors to which the cell is tuned. The center of the movement field is estimated at around [R,Φ] = [37, 20] deg (the yellow-white area). In Figure 1C, the instantaneous firing rates (red) and gaze track velocities (black) are shown for 25 individual trials. Note that for many of these trials there is a strong correlation between the two profiles, even though neural firing rates can be quite noisy. In our earlier work we argued (and showed) that this property betrays tight synchronization of SC bursts in the population during saccades (Goossens and van Opstal, 2006; Goossens and van Opstal, 2012). For this recording, the correlations were *r* > 0.7 for 219/371 trials.

The cell’s movement field can be modeled quantitatively by relating the total number of spikes in the burst to the gaze vector, incorporating the well-established idea that the gaze shift is encoded by a localized (Gaussian) translation-invariant population within the SC motor map (Ottes et al., 1986). Our group has extended this so-called static ensemble-coding model (Ottes et al., 1986; Van Gisbergen et al., 1987) by also accounting for the instantaneous behavior of the firing rates (Goossens and van Opstal, 2006; Goossens and van Opstal, 2012; Van Opstal and Kasap, 2019), linking the cumulative number of spikes of a cell during the gaze shift to the instantaneous intended straight-line displacement of the eye along the trajectory (delayed by the 20 ms neural lead time). The results of this analysis are shown in Figures 1D–F (details are described in Section “Materials and methods”).

### The new model

We recently proposed a conceptual model in which the SC motor map encodes the desired straight gaze trajectory and amplitude by its cumulative spike count, and the saccade kinematics by the total instantaneous SC firing rate. This signal rapidly drives eye and head to the target within a common dynamic gaze-feedback loop (Kasap and van Opstal, 2018b; Van Opstal and Kasap, 2019). In that conceptual model, the SC burst was lumped into a simplified rectangular pulse, rather than by a distributed code of spike trains from a tuned neural population in the motor map. Moreover, the model did not incorporate a neurobiologically plausible mechanism for the influence of eye position on SC activity.

#### Superior colliculus spiking

To explain how the SC neuronal population could control gaze shifts under a variety of initial conditions, we here extend the conceptual scheme by incorporating a two-layer spiking neural network, in which the units displayed similar spiking behaviors as recorded in monkey (Figures 1A,B). In our proposal, the SC motor map issues a feedforward desired straight eye-head gaze trajectory through the dynamic linear accumulation of its individual spike effects to a downstream gaze-feedback comparator (Goossens and van Opstal, 2006; Goossens and van Opstal, 2012). To also account for the influence of initial eye-in-head orientation, we here propose a single neural mechanism by which a feedback oculomotor signal modulates two sensitivity parameters of all units in the motor map to systematically affect their bursting behavior.

#### Superior colliculus movement fields

Note that because of several non-linearities in the downstream circuitry that control the eye- and head-movement kinematics, like a limited oculomotor range, activation of the vestibulo-ocular reflex (VOR; Barnes, 1979; Lefèvre et al., 1992; Tabak et al., 1996; Roy and Cullen, 1998), the varying delay of head-movement onset and resulting head-movement contributions to the gaze shift (Guitton and Volle, 1987; Delreux et al., 1991; Goossens and Van Opstal, 1997; Freedman and Sparks, 2000), the simple linear relationship between SC firing rates and movement kinematics, as shown for head-restrained ocular saccades (Goossens and van Opstal, 2006), might be expected to break down. However, the example of Figure 1D seems to suggest that this relationship may still hold, even in individual cells and for single trials (Figures 1C,F). To better understand these properties, we analyzed the results of our simulations in more detail by quantifying the dynamic SC movement fields of our model units for gaze shifts of different amplitudes from different initial eye orientations and compared the data with results from single-unit recordings of monkey SC cells (Figure 1), as well as with the kinematics and metrics of actual monkey gaze shifts.

## Materials and methods

### Electrophysiological recordings

The monkey experiments (Figures 1, 9B) were performed in the laboratory of Dr. EG Freedman at the Department of Neurobiology and Anatomy, School of Medicine and Dentistry of the University of Rochester, NY, while one of the authors (AJVO) was a visiting scientist. Two rhesus monkeys (P and S) took part in these experiments. They had been trained to follow a briefly flashed visual target with a fast eye-head gaze shift, while single-unit activity was recorded from the left SC (rightward gaze saccades). Animals received a small liquid reward for each successful trial. Details on surgical procedures, training protocols, and experimental setup are described in full detail elsewhere (Quessy and Freedman, 2004; Quessy et al., 2010; Walton and Freedman, 2011). Additional details on the experimental paradigm are provided in the Supplementary material. Experimental procedures and protocols were all approved by the University of Rochester Animal Care and Use Committee, and fully adhered to the National Institutes of Health Guide for the Care and Use of Animals. We recorded from a total of 52 cells, out of which 30 cells could be isolated sufficiently long for a detailed analysis. The movement fields were typically obtained from cells in the caudal SC, where optimal gaze amplitudes ranged from about 30–100°.

**FIGURE 2:**
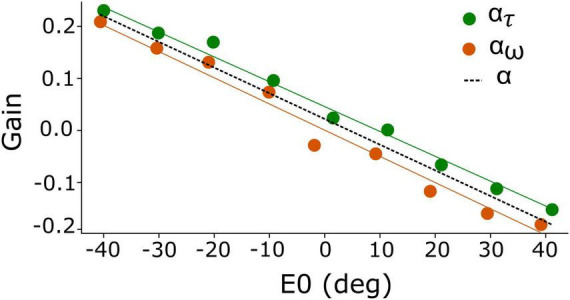
Eye-position dependent values for the gain modulation of the adaptation time constant, α_τ_ (green), and for the excitatory-inhibitory lateral connections, α_*w*_ (orange). Because of the strong resemblance of both gains, their joint behavior is well-described by α (dashed), which yields a linear function of initial eye position. Best fit of Equation 13: a = –0.005 deg^–1^ and b = +0.018.

**FIGURE 3:**
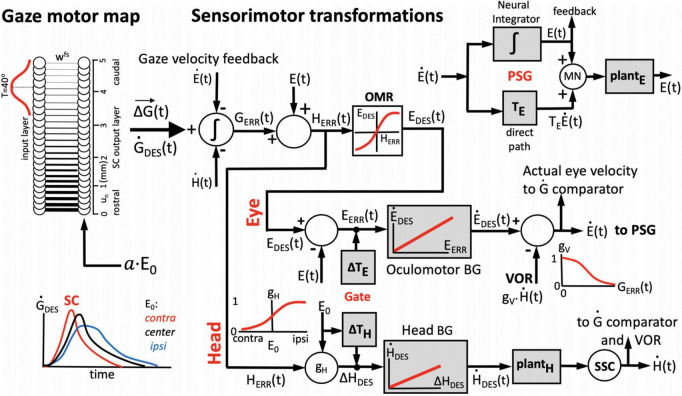
One-dimensional model for the generation of horizontal eye-head gaze shifts. The desired gaze-shift trajectory, ΔG(t), and its associated gaze-velocity profile, ${\dot{\text{G}}}_{\text{DES}}\,\left(\text{t}\right)$, are encoded by the cumulative spike count (Equation 1) and total instantaneous firing rate of the recruited SC population, respectively. The upper part of the control scheme corresponds to the oculomotor system; the lower part to the head-motor system. Initial eye-in-head position, E_0_, modulates the SC units’ firing profiles, as well as the contributions of the eye-and head movements to the gaze shift, ΔE, and ΔH (inset **lower-left**) by influencing their relative timings (Gate). The VOR gain is modulated by the ongoing gaze error, G_*ERR*_(t) (inset, **top-right**). The signal about initial and instantaneous eye position, E(t), is derived from the oculomotor neural integrator in the pulse-step generator (PSG; inset **top-right**). The comparator **(left)** subtracts and integrates the neural estimates of instantaneous eye- and head velocity from the total instantaneous firing rate from the SC motor map, yielding the common gaze-error motor command for eyes and head. See text, for further details.

**FIGURE 4:**
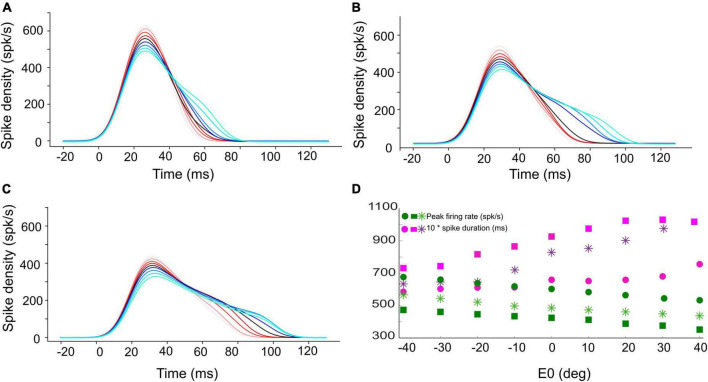
Instantaneous firing rate of the central unit in the population for **(A)** 15°, **(B)** 30°, and **(C)** 45° gaze shifts with the initial eye-in-head position ranging from [–40, +40]° (red to blue colors, respectively). The SC bursts start approximately 20 ms after the start of input current (at *t* = 0). **(D)** The peak firing rate of the central unit (green circles for 15°, green stars for 30°, and green squares for 45° gaze shifts) decreases, while the burst duration (pink symbols) increases, when moving from the more rostral **(A)** to the more caudal **(C)** side, and when the eye moves from the contralateral to the ipsilateral side of the head.

**FIGURE 5:**
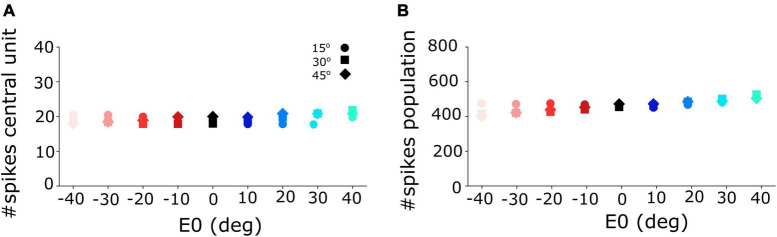
**(A)** Number of spikes emitted by the central unit in the neural population varies only little between 18 and 21 spikes for different initial eye positions and for the three different gaze-shift amplitudes at 15, 30, and 45°. **(B)** The total number of spikes sent by the SC population to the downstream gaze-control circuitry remained virtually invariant across the different initial eye positions and gaze-shift amplitudes.

**FIGURE 6:**
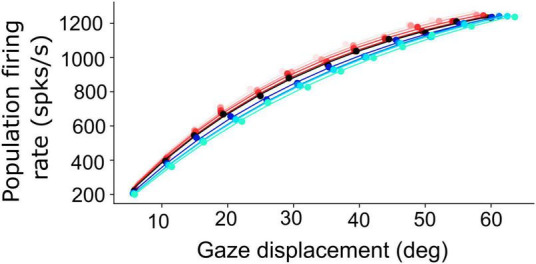
Non-linear, saturating, increase of the planned peak gaze-velocity as function of gaze-shift amplitude as encoded by the SC population firing rate. Results are shown for nine initial eye positions (–40:10:40; different colors) and for 12 sites along the horizontal meridian of the motor map (dots).

**FIGURE 7:**
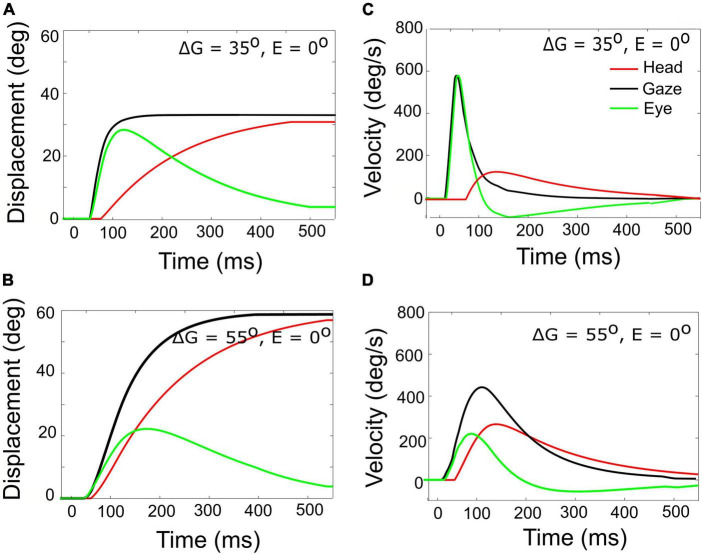
Gaze- (black), head- (red), and eye- (green) displacement (left) and associated velocity profiles (right) for gaze shifts with an amplitude of 35° **(A,C)** vs. 55° **(B,D)**. Note that for the 55° gaze shift, the head onset is earlier, and the head contribution is considerably larger (about 25 vs. 5°), causing the overall gaze velocity to drop considerably, such that it is even slower than the smaller gaze shift. The time axis is referred to burst onset in the SC (20 ms after the input). The additional delay of 10 ms of the gaze shift accounts for the efferent delays in the motor system.

**FIGURE 8:**
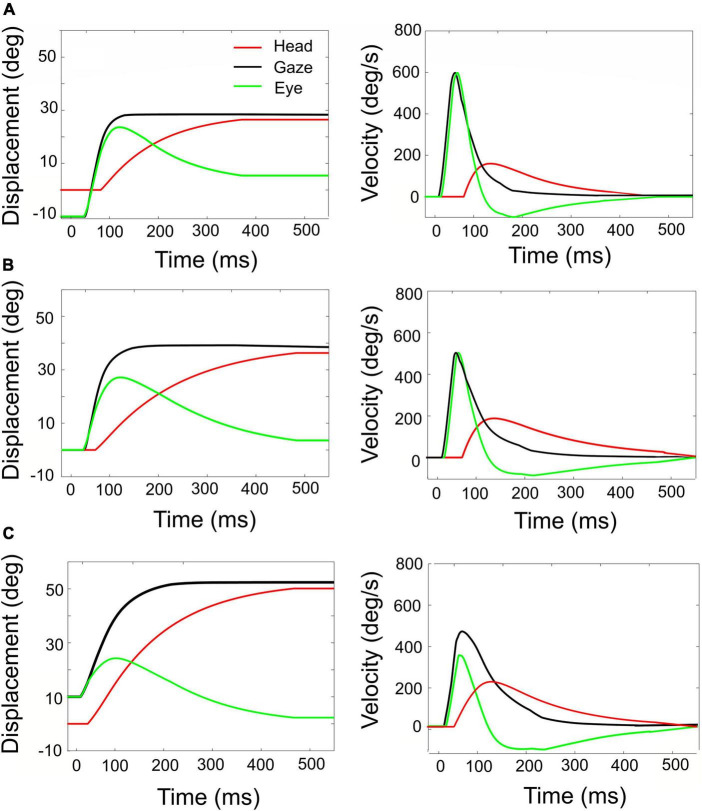
Simulated rightward gaze shifts of 40° amplitude, for three different initial eye-in-head fixations. **(A)** Contralateral initial fixation, at E0 = –10°. **(B)** Eyes at E0 = 0. **(C)** Ipsilateral initial fixation, at E0 = +10°. Left-hand column: eye, head, and gaze trajectories. Right-hand column: eye, head, and gaze-velocities. Note the strong eye-position dependence of the contributions of eye and head to the gaze shift, as well as to the gaze kinematics and head-onset delay. The fastest gaze shift, with the largest eye movement, and smallest and latest head movement is obtained for the eye in the contralateral initial position **(A)**. The slowest gaze shift with the smallest eye movement and the largest head movement is obtained for the eye in the ipsilateral direction **(C)**.

**FIGURE 9:**
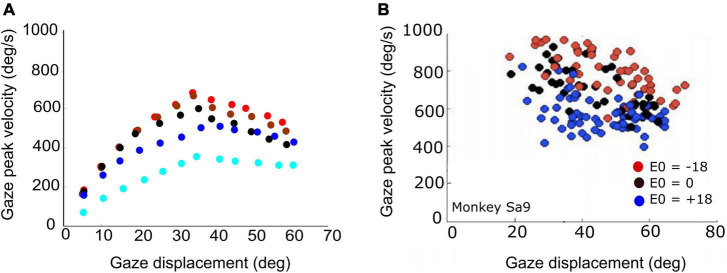
**(A)** Peak gaze velocity as a function of gaze-shift amplitude for five different initial eye-in-head fixations [red (contra): –30°, –10°; black (center): 0°; blue (ipsi): +10°, +30°]. Fastest gaze shifts occur when the eyes fixate contralateral to the gaze shift. Gaze shifts become markedly slower when the eyes fixate ipsilaterally (blue). **(B)** Experimental data from large horizontal gaze shifts between about 20 and 70°, recorded from a macaque monkey for three different initial eye positions (–18°, 0°, 18°; same recording as in Figure 1).

### Network architecture

We modeled the SC gaze motor-map by a one-dimensional two-layer spiking neural network with a cortical input layer, and a layer of SC output units using the Brian2 spiking neural network simulator (Brette and Gerstner, 2005; Goodman and Brette, 2008; Touboul and Brette, 2008). Each layer consists of 200 units, uniformly distributed on 0–5 mm, which corresponds to the SC gaze-motor map midline (horizontal saccades). The units in the input layer all had identical biophysical properties and transformed an externally applied input current into a Gaussian population of spiking activity, which is passed on to the SC units through one-to-one, topography-preserving, synaptic connections. For simplicity, the units in the input layer were assumed not to interact with each other. Furthermore, the spatial-temporal properties of the input current profile were assumed to be invariant for different gaze amplitudes (i.e., the same for all sites in the input layer).

The SC units process the input spikes through their topographically varying intrinsic properties, as described in our earlier work (Kasap and van Opstal, 2017; Alizadeh and Van Opstal, 2022). In short, the biophysical parameters of the SC units, such as their adaptation time constant, their synaptic connection strengths with the input layer, and their lateral excitatory-inhibitory connections, depended in a specific way on their location in the motor map and, as a result, identical spiking activities that arise from the input layer at different locations for different saccade amplitudes will lead to dissimilar responses of the SC units with the appropriate properties: peak firing rate of the central unit in the population decreases, and its burst duration increases, from rostral to caudal sites, while the total number of spikes in the burst remains the same at all sites (e.g., Figures 4A–C; see below).

The neural network’s output represents the desired gaze-shift amplitude by adapting the linear dynamic ensemble-coding scheme for the recruited population as proposed for eye-only saccades (Goossens and van Opstal, 2006; Goossens and van Opstal, 2012) to eye-head gaze shifts. Thus, each single spike from each unit, *n*, in the population is assumed to contribute a small incremental movement, *m*_*n*_, here called the unit’s “spike gaze vector.” In our extended linear ensemble-coding model, the desired gaze trajectory is thus determined by a dynamic cumulative summation of all spike gaze vectors from the activated population:


$$\Delta \,G\,\left(t\right)=\sum_{n=1}^{N_{p\,o\,p}}\sum_{s=1}^{N_{s\,p\,k,n}<t}m_{n}\cdot \delta \,\left(t-\tau_{n,s}\right)\,\text{with}$$

(1) $$\;\,m_{n}=\kappa \cdot A\,\left[e\,x\,p\,\left(\frac{u_{n}}{B_{u}}\right)-1\right]$$

in which *t* is the current time, δ(*t*−τ_*n*,*s*_) represents a spike of unit *n* at time *τ_*n,s*_*, *N*_*pop*_ is the total number of active units in the population, and *N*_*spk,n*_ *< t* is the total number of spikes fired by unit *n* up to time *t*. The spike gaze vector depends exclusively on its rostral-caudal coordinate, *u*_*n*_, with *u*_*n*_ ∈ [0−5] mm encoding the horizontal gaze-saccade amplitude in deg, and κ is a fixed scaling factor that only depends on the assumed cell density (in number neurons/mm). This scaling factor was calibrated for a horizontal saccade of 15°. The SC afferent mapping parameters (here: *A* = 3.0°, and *B*_*u*_ = 1.4 mm) were adopted from monkey microstimulation data (Robinson, 1972; Ottes et al., 1986; Van Gisbergen et al., 1987) (see also the Supplementary material, for more details).

#### Neural dynamics

The network units were generated with a time resolution of 0.01 ms. We implemented adaptive exponential integrate-and-fire (AdEx) units, which enables realistic bursting dynamics. The AdEx neuronal unit is a simplification of the biophysical Hodgkin–Huxley model, as it contains only two state variables: the membrane potential, *V(t)*, and the adaptation current, *q(t)*. The dynamics of the AdEx model are thus determined by two coupled non-linear differential equations in which all parameters have a clear physiological interpretation:


$$C\,\frac{d\,V_{n}}{d\,t}=-g_{L}\,\left(V_{n}-E_{L}\right)+g_{L}\,\eta \,e\,x\,p\,\left(\frac{V_{n}-V_{T}}{\eta }\right)-q_{n}$$

(2) $$+I_{s\,y\,n,n}\,\left(t\right)$$

(3) $$\tau_{q,n}\cdot \frac{d\,q_{n}}{d\,t}=a\,\left(V_{n}-E_{L}\right)-q_{n}$$

Here, *C* is the membrane capacitance, *g*_*L*_ is the leak conductance, *E*_*L*_ is the leak-reversal potential, η is a slope factor, *I*_*syn,n*_ is the unit’s total synaptic input current, *τ_*q*_* is the adaptation time constant, *a* is the subthreshold adaptation constant, and *V*_*T*_ the threshold potential.

In the SC model units, two biophysical parameters depended on the location of the neuron (*u*_*n*_) in the motor map, and thus determined the SC bursting properties: the adaptation time constant, τ_*q,n*,_ and the synaptic input current, *I*_*syn,n*_. The other parameters, *C*, *g*_*L*_, *E*_*L*_, η, *V*_*T*_, and *a*, were all location-independent and were optimized such that they ensured appropriate bursting behavior (see Supplementary Table 1, for the values of all parameters). Optimal parameter values for the AdEx units, and for all network connections, were obtained from a brute-force algorithm, as there exists no analytical solution for the system.

A spike is triggered whenever the membrane potential, *V(t)*, rapidly grows to infinity. In practice, however, we set the spiking threshold at *V*_*T*_. The slope factor determines the sharpness of the threshold and implements a smooth spike initiation zone instead of a strict all-or-nothing spiking threshold. For each spiking event at time, τ, the integration of the equation is reset to its resting potential, *V*_*r*_, and the adaptation current, *q*, is increased by *b* to implement the spike-triggered adaptation:


(4) $$V_{n}\,\left(\tau \right)\to V_{r},q_{n}\,\left(\tau \right)\to q_{n}\,\left(\tau \right)+b$$

### Synaptic function of the superior colliculus neural model

An SC unit in the motor map receives its total input from spiking activity from all surrounding collicular units through conductance-based synapses from the excitatory and inhibitory synaptic transmission, and from spiking activity of the input layer units, which in turn are stimulated by an externally applied input current.

The one-to-one connection strengths between input layer and SC units were location-dependent according to:


(5) $$w^{F\,S}=w_{m\,a\,x}^{F\,S}+\left(n-1\right)\cdot \frac{w_{m\,i\,n}^{F\,S}-w_{m\,a\,x}^{F\,S}}{N-1}$$

with *N* = 200 the total number of units in the SC layer, *n* the unit number, and [$w_{m\,i\,n}^{F\,S},$$w_{m\,a\,x}^{F\,S}$] were set at [4, 10] nS, respectively.

The total excitatory-inhibitory synaptic input to an SC unit is given by:


(6) $$I_{s\,y\,n,n}\,\left(t\right)=g_{n}^{e\,x\,c}\,\left(t\right)\,\left(E_{e}-V_{n}\,\left(t\right)\right)+g_{n}^{i\,n\,h}\,\left(t\right)\,\left(E_{i}-V_{n}\,\left(t\right)\right)$$

where $g_{n}^{e\,x\,c}$ and $g_{n}^{i\,n\,h}$ are the excitatory and inhibitory synaptic conductances of unit *n*, and *E*_*e*_ and *E*_*i*_ are the excitatory and inhibitory reversal potentials, respectively (for their values, see Supplementary Table 1).

The excitatory conductances respond dynamically to the activity of the collicular units and the external cortical input. The conductance increases with each presynaptic spike from the excitatory inputs from nearby SC units and for each spike from the cortical input unit at the same location, and it decays exponentially with time constant τ_*exc*_ according to:


$$\tau_{e\,x\,c}\,\frac{d\,g_{n}^{e\,x\,c}}{d\,t}=-g_{n}^{e\,x\,c}+\tau_{e\,x\,c}\,w_{n}^{F\,S}\,\sum_{s}^{N_{s\,p\,k}^{I\,n\,p\,u\,t}}\delta \,\left(t-\tau_{n,s}\right)$$

(7) $$\;+\tau_{e\,x\,c}\,\sum_{i}^{N_{p\,o\,p}^{S\,C}}w_{i,n}^{e\,x\,c}\,\sum_{s}^{N_{p\,o\,p}^{S\,C_{i}}}\delta \,\left(t-\tau_{i,s}\right)$$

Similarly, the inhibitory conductance increases with each presynaptic spike from all SC units with an inhibitory connection to unit *n*, after which it decays exponentially with time constant τ_*inh*_:


(8) $$\tau_{i\,n\,h}\,\frac{d\,g_{n}^{i\,n\,h}}{d\,t}=-g_{n}^{i\,n\,h}+\tau_{i\,n\,h}\,\sum_{i}^{N_{p\,o\,p}^{S\,C}}w_{i,n}^{e\,x\,c}\,\sum_{s}^{N_{s\,p\,k}^{S\,C_{i}}}\delta \,\left(t-\tau_{i,s}\right)$$

In Equations 7, 8, $w_{n}^{F\,S}$is the synaptic strength between input layer unit *n* and SC unit *n*; $w_{i,n}^{i\,n\,h}$ and $w_{i,n}^{e\,x\,c}$ are the intra-collicular excitatory and inhibitory lateral connection strengths between units *i* and *n*, while τ_*x,s*_ is the timing of the presynaptic spike in the spike train *s*, δ(*t*−τ_*x*,*s*_), projecting from unit *x* to unit *n.*

### Saccade target representation by external input current

We provided a desired target vector, T, to the network by an external input current evoking a population activity centered around the image point, *u*_*T*_ (Equations 9, 15). The central unit in the input population receives the maximum input activation current, *I_0_(t)*, while the other units in the input layer are stimulated by current strengths that decay as a Gaussian with distance from *u*_*T*_. The spatial-temporal external input current was thus described by a separable spatial-temporal function on the input units by:


$$I_{e\,x\,t}\,\left(u_{n},t\right)=I_{0}\,\text{exp}\,\left(-\frac{{||u_{n}-u_{T}||}^{2}}{2\,\sigma_{p\,o\,p}^{2}}\right)\cdot t^{\gamma }\,\exp\left(-\beta \,t\right)$$

(9) for⁢t≥0

where *u*_*n*_ is the anatomical position of unit *n* in the input map, *σ_*pop*_* specifies the size of the recruited input population, *t* is time (in s), *u*_*n*_ is the location of unit *n* (mm), and *I*_0_ is the maximum input amplitude (pA). The time-dependent term is a gamma function, characterized by γ (skewness, dimensionless) and β (measure for the inverse input duration, in s^–1^).

### Superior colliculus units and influence of initial eye-in-head position

We have shown previously that the adaptation time constant (τ_*q*_) systematically affects the peak-firing rate and burst-duration of the SC units, while the synaptic projection strengths between the input layer and the SC layer mainly affect the SC peak firing rate (Kasap and van Opstal, 2017; Alizadeh and Van Opstal, 2022).

As the adaptation time constant and synaptic connection strengths between the two layers systematically decrease from the rostral to the caudal pole of the SC motor map, rostral units generate small saccades with high-frequency, short-lasting bursts of activity for their preferred saccade, while units at caudal sites, associated with large saccades, have lower peak firing rates and longer burst durations (e.g., Figure 4). In line with neural recordings, however, the total number of spikes from the bursts in the population is invariant to the saccade amplitude, and even to the saccade kinematics: slow and fast saccades of the same amplitude are associated with different firing rates and burst durations but are encoded by the same number of spikes (Goossens and van Opstal, 2006; Goossens and van Opstal, 2012). This invariance was achieved in the model by co-tuning the adaptation time constant (τ_*q*_) and lateral connection parameters (Kasap and van Opstal, 2017; Alizadeh and Van Opstal, 2022).

#### Model extensions

To extend the model to the control of eye-head gaze shifts, we included a modulatory signal proportional to the initial eye-in-head orientation, *E*_0_, that influences the firing properties of the SC units, with only a minimum effect on the total number of population spikes. As observed in single-unit recordings (Van Opstal and Kasap, 2019; Figure 1), ipsilateral eye positions with respect to the target direction lead to a decrease in the neural peak-firing rate and an increase in burst duration compared to the straight-ahead eye direction, whereas a contralateral eye position leads to the opposite effect: higher peak firing rates with shorter burst durations (see Section “Introduction”).

The model’s adaptation time constant, *τ_*n*_*, as function of the map index, *n*, and eye-position dependent gain, α_τ_, is thus described by:


(10) $$\tau_{n}\,\left(E_{0}\right)=\left(1+\alpha_{\tau }\,\left(E_{0}\right)\right)\cdot \left(\tau_{m\,a\,x}+\left(n-1\right)\cdot \frac{\tau_{m\,i\,n}-\tau_{m\,a\,x}}{N-1}\right)$$

with *N* = 200 the total number of units in the SC layer, *n* the unit number, and [τ_*min*_, τ_*max*_] were set at [30, 60] ms, respectively. Note that for $\alpha_{\tau }=0$ the time constant is identical to the Kasap and van Opstal (2017) model.

Effectively, SC units receive both excitatory and inhibitory potentials from units endowed with different adaptation time constants, firing rates, and reversal potentials through the lateral connections (Equations 11a,b). We modeled the latter by a Mexican hat-type connection scheme, where the net synaptic effect is given by the difference between two Gaussians (Touboul and Brette, 2008; Kasap and van Opstal, 2017; Alizadeh and Van Opstal, 2022). We showed in our previous work that because of this soft winner-take-all organization, the central unit in the neural population (the “winner”) synchronizes all other bursts in the population (Goossens and van Opstal, 2012; Kasap and van Opstal, 2017; Alizadeh and Van Opstal, 2022).

The lateral connections were modulated by a similar eye-position dependent gain in the following way:


$$w_{i,n}^{e\,x\,c}\,\left(E_{0}\right)=S_{n}\,\left(1+\alpha_{w}\,\left(E_{0}\right)\right)\cdot {\bar{w}}_{e\,x\,c}\,\text{exp}\,\left(-\frac{{||u_{i}-u_{n}||}^{2}}{2\,\sigma_{e\,x\,c}^{2}}\right)$$

(11a) for⁢n≠i

(11b) $$w_{i,n}^{i\,n\,h}\,\left(E_{0}\right)=S_{n}\,\left(1+\alpha_{w}\,\left(E_{0}\right)\right)\,\left(1-{\bar{w}}_{i\,n\,h}\,\text{exp}\,\left(-\frac{{||u_{i}-u_{n}||}^{2}}{2\,\sigma_{i\,n\,h}^{2}}\right)\right)$$

(11b) for⁢n≠i

where ${\bar{w}}_{e\,x\,c}=0.16\,\text{nS}$ and ${\bar{w}}_{i\,n\,h}=1.15\,\text{nS}$ are fixed excitatory and inhibitory weight parameters. The scaling parameter *S*_*n*_ is the map-location-dependent gain, making the lateral interaction scheme site-dependent:


(12) $$S_{n}=1-0.04.u_{n}^{2}$$

The lateral inhibitory and excitatory connection strengths ($w_{i,n}^{e\,x\,c},w_{i,n}^{e\,x\,c}$) decrease from the rostral to the caudal zone, which resulted to mainly influence the shape of the non-linear main-sequence relationship of the model’s saccades between their amplitude and peak eye velocity.

### Network tuning

We employed brute-force search algorithms to find suitable values for the eye-position dependent gains of the adaptation time constant (Equation 10), the lateral inhibitory and excitatory weights (Equations 11a,b), the intrinsic properties of the AdEx equations of the SC units (Equations 2, 3), and the feedforward projections, $w_{n}^{F\,S}$ (Equation 5). The intrinsic biophysical parameters of the AdEx model (Supplementary Table 1) were optimized by systematically varying τ_*q,n*_, in combination with $w_{n}^{F\,S}$ in a linear way with *u*_*n*_. Optimal values of the biophysical parameters are identical to those in our previous study for E_0_ = 0° (Kasap and van Opstal, 2017; Alizadeh and Van Opstal, 2022).

We initially set out to modulate the adaptation time constant and lateral connections independently as function of eye position, by α_τ_(E_0_) and α_*w*_(E_0_), respectively (Equations 10, 11a,b). The result of our brute-force tuning is shown in Figure 2. Remarkably, the optimal values for the two parameters appeared to vary nearly linearly with *E*_0_ and were highly correlated. We therefore lumped the two gains into a single modulation gain, hence on denoted by *α(E_0_)*. Best results were obtained for a simple affine relation (Figure 2, dashed line):


(13) $$\alpha \left(E_{0}\right)=a\cdot E_{0}+b$$

Note that α(*E*_0_) was taken the *same* for all SC units. Thus, the eye-position signal was distributed uniformly across the motor map.

### Desired eye-movement trajectories

The population activity in the SC motor map encoded desired eye-head gaze shifts by the dynamic linear ensemble-coding scheme for the one-dimensional efferent motor map (Equation 1). The resulting instantaneous desired gaze-displacement trajectory, *ΔG(t)*, was interpolated with a Savitzky–Golay filter to compute smooth instantaneous desired gaze velocity profiles.

### Superior colliculus unit movement fields

To analyze the movement-field properties of the SC units, we counted for each unit the cumulative number of spikes in the burst during all horizontal gaze shifts with amplitudes between 5 and 55° in 2° steps, and for three initial eye-in-head orientations (−20, 0, +20°). We first fitted the units’ *static* movement-field functions for gaze saccades, by adapting the original quantitative model of Ottes et al. (1986) and Van Gisbergen et al. (1987) for eye-only saccades to eye-head gaze shifts and included a potential effect of the initial eye-in-head orientation on the number of spikes in the burst (Van Opstal et al., 1995). Thus, the total number of spikes, *N*, of an SC cell was described by:


(14) $$N\,\left(△\,G,E_{0},u_{n}\right)=N_{0}\,\left(1+\epsilon \cdot E_{0}\right)\cdot \text{exp}\,\left(-\frac{{\left(u_{n}-u_{0}\right)}^{2}}{2\,\sigma_{P}^{2}}\right)$$

The static movement-field function predicts the total number of spikes in the SC burst to an arbitrary gaze shift for every neuron *n* in the map and for each initial eye position. In our 1D model, the static movement field has four free parameters: *N*_0_ is the number of spikes in the burst for the unit’s optimal saccade from straight ahead, *u*_0_ (in mm) is the optimal saccade coordinate in the SC motor map for the population, ϵ (in #spikes/deg) is the measured eye-position sensitivity of the unit, and *σ_*p*_* (in mm) quantifies the unit’s tuning width (spatial extent of the gaze-shift field as point-mapped on the motor map). Finally, *u*_*n*_ is the anatomical SC coordinate of unit *n* for its own optimal gaze shift, and is obtained by the (1D) afferent mapping function (Van Gisbergen et al., 1987):


(15) $$u_{n}=B_{u}.\ln\left(\frac{△\,G_{n}+A}{A}\right)$$

where B_*u*_ = 1.4 mm and A = 3.0° (e.g., Figures 1B,E; Ottes et al., 1986; Van Gisbergen et al., 1987).

Next, we quantified the neuron’s instantaneous firing profile by the *dynamic* movement-field function that predicts how the cumulative number of spikes in the burst of unit *n* evolves as a function of time during each desired straight gaze-displacement (Goossens and van Opstal, 2006; Van Opstal and Kasap, 2019). We here extended our original concept for eye-only saccades by including the gain-influence of initial eye-in-head position, E_0_. As a consequence of the dynamic ensemble-coding concept (Equation 1), the cell’s dynamic movement field is predicted to behave according to the following linear relation:


(16) $$C\,S\,\left(△\,G,E_{0},u_{n},t\right)=\frac{N\,\left(△\,G,E_{0},u_{n}\right)}{△\,G}\cdot △\,G\,\left(t+△\,T_{G}\right)$$

with △*G*(*t*+△*T*_*G*_) the desired straight trajectory (corrected for the fixed neural lead time, here taken as 10 ms), which increases monotonically from 0 to the final amplitude, △*G*. The time-independent factor in Equation 16, *N*(△*G*,*E*_0_,*u*_*n*_)/△*G*, is the expected slope of the linear dynamic phase-relation between the ongoing (delayed) gaze shift and the expected cumulative number of spikes of the cell (see, e.g., Figures 1D,F). This slope is proportional to the number of spikes from the static movement field (Equation 14) and is inversely related to the gaze-shift amplitude (Van Opstal and Kasap, 2019).

### Sensorimotor transformation for generating eye-head gaze shifts

We implemented a 1D sensorimotor model for the generation of eye-head gaze shifts, in which the SC cells act as a common gaze-shift command to reorient the eyes and head toward the target (Goossens and Van Opstal, 1997; Kasap and van Opstal, 2018b). The population of recruited SC cells encodes the dynamic desired straight gaze trajectory, *ΔG(t)*, through Equation 1 (Goossens and van Opstal, 2006; Goossens and van Opstal, 2012). The parameters of the network units are influenced by the distributed initial eye-position signal (Equations 10–12). The schematic outline of our computational model is shown in Figure 3.

The firing rates of the population of recruited SC cells effectively encode a desired dynamic gaze-velocity profile, ${\dot{G}}_{D\,E\,S}\,\left(t\right)$ by the summed instantaneous firing rates of all neurons. Inspired by Scudder’s model for eye-only saccades (Scudder, 1988), the instantaneous gaze-motor error follows from the ongoing difference between the cumulative integral (spike count) of the desired gaze velocity signal from the SC and the true gaze velocity constructed from feedback of the downstream oculomotor and head-motor burst controllers:


(17) $$G_{E\,R\,R}\,\left(t\right)=\int_{O\,N}^{t}\left({\dot{G}}_{D\,E\,S}\,\left(t\right)-\dot{E}\,\left(t\right)-\dot{H}\,\left(t\right)\right)\,dt$$

#### Oculomotor system

Note that the gaze-error of Equation 17 may extend far beyond the mechanical, head-centered, oculomotor range (OMR; upper section of Figure 3). To prevent this from happening, the oculocentric gaze error is first transformed into a craniocentric (eye-in-head) error by adding a neural estimate of current eye position. The latter is obtained from the oculomotor neural integrator within the pulse-step generator (PSG) of Figure 3 (Robinson, 1973; Cannon and Robinson, 1987):


(18) $$H_{E\,R\,R}\,\left(t\right)=G_{E\,R\,R}\,\left(t\right)+E\,\left(t\right)$$

The ongoing head-centered error of Equation 18 drives both the oculomotor and head-motor control systems (Goossens and Van Opstal, 1997; Kasap and van Opstal, 2018b). To keep eye position within the mechanical limits of the head-centered OMR (here set between −30 and +30°), H_*ERR*_(t) for the eye was constrained by a soft limiter that yields the actual desired eye-in-head position:


(19) $$H_{D\,E\,S}\,\left(t\right)=30\cdot \tanh\left(\beta \cdot H_{E\,R\,R}\,\left(t\right)\right)$$

with β = 0.03 deg^–1^. Note that this desired eye position is a time-varying signal that changes during the execution of the gaze shift. The instantaneous eye motor-error is derived by subtracting current eye position:


(20) $$E_{E\,R\,R}\,\left(t\right)=H_{D\,E\,S}\,\left(t\right)-E\,\left(t\right)$$

This eye-error drives the *linear* oculomotor burst generator, with a fixed gain of B_*E*_ = 60 s^–1^, to generate a desired eye-velocity signal (Goossens and van Opstal, 2006; Goossens and van Opstal, 2012):


(21) $${\dot{E}}_{D\,E\,S}\,\left(t\right)=B_{E}\cdot E_{E\,R\,R}\,\left(t\right)$$

Note that a linear burst generator is a unique feature of our model, as it delegates the origin of the non-linear saturation of peak eye velocity (main-sequence kinematics, see Introduction) to the upstream collicular controller (Goossens and van Opstal, 2006).

Finally, the true eye-velocity command for the oculomotor neurons, neural integrator, and eye plant (the final common PSG path, Figure 3; Robinson, 1973) is obtained by combining the desired velocity signal of Equation 21 with the gain-modulated VOR (Whittington et al., 1984; Laurutis and Robinson, 1986; Tomlinson and Bahra, 1986b). The latter is driven by the true head velocity, and its gain, *g*_*V*_, is determined by instantaneous gaze error:


(22) $$\dot{E}\,\left(t\right)={\dot{E}}_{D\,E\,S}\,\left(t\right)-g_{V}\,\left(G_{E\,R\,R}\,\left(t\right)\right).\dot{H}\,\left(t-△\,T_{H}\right)\,\mathrm{where}$$

$$g_{V}\,\left(G_{E\,R\,R}\right)=\left(1-\tanh\left(0.03\cdot G_{E\,R\,R}\right)\right)$$

The VOR gain is close to 1.0 (i.e., fully engaged) when the gaze error is small (the eye is on target), and rapidly falls to zero (i.e., inactive) for large gaze errors (e.g., at the start of the gaze shift) (Laurutis and Robinson, 1986; Guitton and Volle, 1987; Goossens and Van Opstal, 1997), allowing the eyes to move in the direction of the goal at optimal speed.

For the ocular plant we took the simple second-order linear low-pass filter given by


(23) $$P_{E\,Y\,E}\,\left(\tau \right)=\frac{1}{T_{E\,1}-T_{E\,2}}\,\left(\exp\left(-\tau /T_{E\,1}\right)-\exp\left(-\tau /T_{E\,2}\right)\right)$$

with *T*_*E1*_ = 200 ms and *T*_*E2*_ = 20 ms, respectively.

#### Head-motor system

In parallel, the head-motor system (lower section of Figure 3) is driven by the head-motor error of Equation 18, scaled by a gain that depends on initial eye position:


(24) $$△\,H_{D\,E\,S}\,\left(t-△\,T_{H}\right)=g_{H}\,\left(E_{0}\right).H_{E\,R\,R}\,\left(t\right)$$

$$\text{with}\,g_{H}\,\left(E_{0}\right)=0.5\cdot \left(1+\tanh\left(0.05\cdot E_{0}\right)\right)$$

Importantly, the head-onset delay, *ΔT_*H*_*, depends on initial eye orientation and on the (absolute) gaze-shift amplitude. We here modeled this effect by a simple bi-linear function, thereby assuming a similar mechanism of initial eye orientation on the head movement as on the SC units (Equation 10–12; Figure 2):


(25) $$△\,T_{H}=70-0.72\cdot \left|△\,G\right|-E_{0}\,\text{ms}$$

In this way, the delay increases for contralateral eye positions (E_0_ < 0) and decreases for large gaze shifts and ipsilateral eye positions. The desired head velocity was subsequently generated by a linear head-burst generator with a fixed gain of B_*H*_ = 20 s^–1^:


(26) $${\dot{H}}_{D\,E\,S}\,\left(t-△\,T_{H}\right)=B_{H}.△\,H_{D\,E\,S}\,\left(t-△\,T_{H}\right)$$

The actual head velocity, as measured by the vestibular canals, follows passing the desired head-velocity command through a sluggish second-order low-pass filter as a simplified model for the head-motor plant:


$$\dot{H}\,\left(t-△\,T_{H}\right)=\int_{0}^{\infty }P_{H\,E\,A\,D}\,\left(\tau \right)\,{\dot{H}}_{D\,E\,S}\,\left(t-△\,T_{H}-\tau \right)\,d\tau \,\text{and}$$

(27) $$P_{H\,E\,A\,D}\,\left(\tau \right)=\frac{1}{T_{H\,1}-T_{H\,2}}\,\left(\exp\left(-\tau /T_{H\,1}\right)-\exp\left(-\tau /T_{H\,2}\right)\right)$$

where we took T_*H1*_ = 250 ms, and T_*H2*_ = 150 ms.

In the simulations, presented below, we varied the initial eye orientation E_0_ between [−40*^o^*, +40*^o^*] and gaze-shift amplitudes ΔG between 1 and 60°.

## Results

### Eye-position influence on superior colliculus activity

Figures 4A–C shows the burst profiles for three example SC units for their optimal gaze shifts with the eye in nine different initial positions (contralateral: reddish lines; ipsilateral: bluish lines) from −40 to +40° in 10° steps. Note the systematic increase of burst duration (purple symbols) and the corresponding decrease of the peak firing rate (by about 20%; green symbols) as the eye-in-head position varies from the contralateral to the ipsilateral side (Figure 4D).

Figure 5A shows that the central units in the three different neural populations emitted approximately the same number of spikes (*N*_*spk,n*_ ≈ 20) for the three desired gaze shifts (different symbols), and for all eye-in-head orientations, despite the substantial changes in the peak firing rates and burst durations of their firing profiles (Figure 4). Similarly, the total number of spikes of the three neural populations remained practically invariant at about 450 spikes for the different gaze-shift amplitudes across all eye-in-head positions (Figure 5B).

Figure 6 shows how the planned peak gaze velocity (given by the total summed peak firing rate of the population) varies with the desired gaze-shift amplitude (from Equation 1), and with the changes in initial eye-orientation (different colors; black: default condition, *E*_0_ = 0°). The planned kinematics show the typical saturation observed for head-restrained eye saccades (Bahill et al., 1975; Goossens and van Opstal, 2006; Goossens and van Opstal, 2012), with a slight modulation of the intended peak velocity because of the eye-position gain (Equation 12). Note, however, that in the case of eye-head gaze shifts, these planned gaze kinematics may become dissociated from the true gaze kinematics, because the SC units in the model do not sense any of the associated changes in the eye- vs. head-movement contributions that are determined in the downstream feedback circuitry of the model (Figure 3).

### Eye-head coordination

A horizontal saccadic eye-head gaze shift (ΔG) is composed of the linear sum of the instantaneous eye-in-head and head-on-neck orientations: *ΔG(t) = ΔE(t)* + *ΔH(t)*. The contribution of the head movement during the gaze shift has a strong effect on the gaze kinematics. This point is illustrated in Figure 7, which exemplifies two gaze shifts: one of 35° (Figures 7A,C) and one of 55° (Figures 7B,D).

Both gaze shifts start with the eye and head directed at straight ahead (i.e., E_0_ = H_0_ = 0°). For the larger gaze saccade, the head movement starts earlier after the eye-movement onset than for the smaller gaze saccade (Equation 25). Because of the much larger head contribution in the latter case (about 25 vs. 5° at gaze-shift offset), the 55° gaze shift is considerably slower than the 35° gaze shift, and therefore breaks with the monotonically increasing main-sequence relationship for eye saccades (Bahill et al., 1975; also Figure 6).

The influence of initial eye position on the kinematics of simulated gaze shifts, and on the associated head- and eye movements is further illustrated in Figure 8, for fixed-amplitude 40° gaze saccades, generated from three initial eye-in-head orientations: contralateral (Figure 8A), aligned (Figure 8B), and ipsilateral (Figure 8C). Note the different head-movement contributions during these gaze shifts and the associated changes in the gaze-velocity profiles for the different initial conditions. As initial eye-in-head position moves from contralateral (A) to ipsilateral (C), the relative eye movement gets smaller and slower and the head contribution to the gaze shift increases because it starts earlier. Consequently, the overall gaze shift becomes slower (Guitton and Volle, 1987; Goossens and Van Opstal, 1997; Freedman and Sparks, 2000).

The actual main-sequence relation for the model’s gaze shifts (amplitude vs. peak-velocity) for gaze amplitudes between 5 and 60° is shown in Figure 9A for five different eye-in-head orientations: aligned (E_0_ = 0; black), eye contralateral (at −10, or −30°; red and brown symbols), and eye ipsilateral (+10°, +30°; dark and light blue symbols) of the target. Note that for small gaze shifts (*ΔG* < 40°), gaze velocity systematically increases with amplitude, just like for head-fixed eye saccades. However, for larger gaze shifts (*ΔG* > 40°), the peak gaze velocity starts to drop considerably with increasing gaze-shift amplitude. This effect was highly significant for both the contralateral and centered eye positions, and less strong for the ipsilateral eye orientations. Similar behavior has been reported for monkey gaze shifts (Tomlinson and Bahra, 1986a; Freedman, 2001, 2008; Gandhi, 2012; Van Opstal and Kasap, 2019). Figure 9B shows a representative example of data from a monkey making large horizontal gaze shifts from three different initial eye orientations (Supplementary Figure 1).

According to our model, this remarkable property is due to two factors: first, for large gaze shifts, the planned eye movement will rapidly approach the oculomotor range so that the eye-in-head velocity will start to plateau (Guitton and Volle, 1987; Goossens and Van Opstal, 1997; Freedman and Sparks, 2000). Second, at increasing gaze amplitudes the contribution of the (slower) head movement increases too (Figure 7; Equation 24). As a result, the head velocity will increasingly dominate the gaze velocity profile when the gaze-shift amplitude increases. The effect is least pronounced for the far-ipsilateral condition because in that case the head trajectory already coincides nearly fully with the eye trajectory. As a result, the gaze shift is already maximally influenced by the head.

### Static and dynamic superior colliculus movement fields

To link the firing patterns of the spiking neural network (Figures 4–6) to the model’s output (eye- and head movement trajectories; Figures 7–9), we next analyzed the static and dynamic movement-field properties of the model SC units for the different gaze-shifts.

**FIGURE 10:**
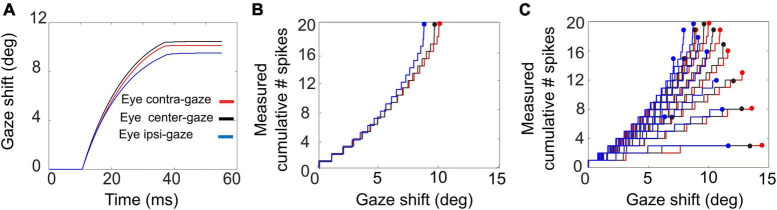
Gaze shifts of 10° amplitude without a head movement, for three different initial eye-in-head fixations: E_0_ = –10° (red), 0° (black), +10° (blue). **(A)** Gaze trajectories. Note the different saccade speeds with varying eye position. **(B)** Phase trajectories of the cumulative number of spikes of the central unit as function of ongoing gaze displacement (back-shifted by 10 ms). Note that the curves superimpose although the gaze kinematics differ substantially. **(C)** Cumulative number of spikes in the burst vs. ongoing (BACK–SHIFTED) gaze displacement for all saccades (7–15°) into the cell’s movement field (cf. Figure 1D).

The feedforward encoding of the gaze kinematics (by the population firing rate; Figures 3, 6) is expected to become dissociated from the actual gaze kinematics (Figures 7–9) when the head starts to contribute to the gaze shift, since the SC units in our model have no access to a head-movement signal, which in turn strongly determines the gaze kinematics. Without a head movement, however, the dynamic linear ensemble-coding model of Equation 1 predicts a one-to-one relationship between the eye-saccade velocity and the population firing rate (Goossens and van Opstal, 2006), as well as the straight-line relations between the cumulative number of spikes and the instantaneous eye displacements (dynamic movement fields). Figure 10 illustrates these properties for 10*^o^* gaze shifts without a head movement (i.e., we set g_*H*_ = 0 in the simulations) generated from three different initial eye positions.

Although the gaze kinematics are considerably affected by the initial eye orientation (Figure 10A), the phase curves that relate the cumulative number of spikes of the central SC unit to the instantaneous change in gaze for the optimal gaze amplitude (aligned by ΔT_*G*_ = 10 ms) are quite similar (Figure 10B). This behavior is a direct consequence of the linear ensemble-coding concept (Equation 1), in which each spike of each unit contributes a fixed gaze displacement vector. Note, however, that since the ordinate represents the output of a single neuron, whereas the abscissa is the output of the total neural population, the tight resemblance of the three phase curves should be understood from the high level of synchronization of the bursts among all recruited units. The latter is caused by the soft winner-take-all interactions in the motor map (Kasap and van Opstal, 2017). Figure 10C shows the phase curves for all gaze saccades into the unit’s movement field for three initial eye positions. The dots at the end of the curves correspond to the final gaze displacement and total number of spikes (cf. with Figure 1D). These dots should follow the static movement field of Equation 14, where the total number of spikes depends on gaze amplitude (between about 6 and 15°) and initial eye position. Note that the phase curves have different slopes for each gaze amplitude, like in Figure 1D. For eye-only saccades, neural recordings have shown that each of these lines may be predicted by the dynamic movement-field description of Equation 16 (Goossens and van Opstal, 2006). It is not a-priori obvious, however, that the same should hold also for our eye-head gaze-control system with its inherent non-linearities (Figure 3).

**FIGURE 11:**
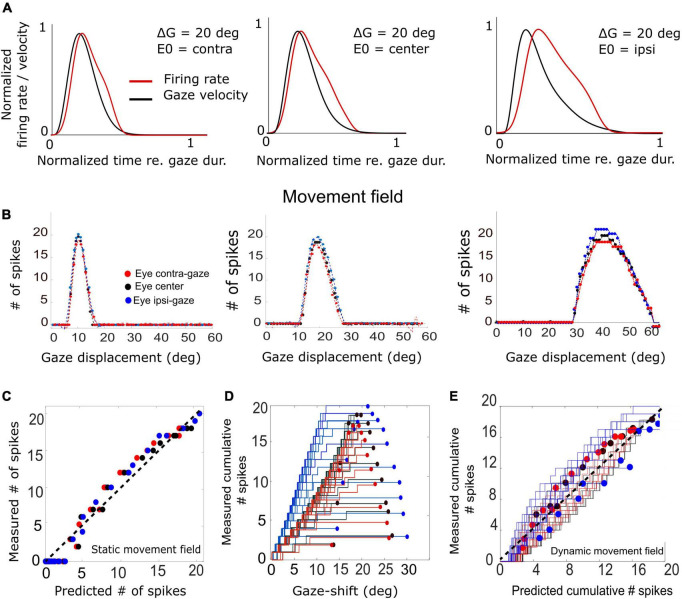
**(A)** Three example trials demonstrating the relation between the unit’s firing-rate profile and the instantaneous gaze velocity for three initial eye positions in a 20° gaze-shift. For ease of comparison, both variables were normalized to gaze duration and to their maxima. The correlation is highest for the contralateral situation (*r* = 0.92) and decreases for the ipsilateral condition (*r* = 0.69). **(B)** Plot of the three static movement fields for units with their optimal saccades at 10, 20, and 40°, respectively (Equation 14) for three different initial eye positions. Note that the movement fields vary little, but systematically, with initial eye position. **(C)** The static gain-field model (Equation 14) captures the data well for all gaze shifts and initial conditions for the unit with its optimum at 20° (see also Table 1). **(D)** Phase trajectories of the cumulative number of spikes of the unit as a function of ongoing gaze displacement along the straight gaze vector (dynamic movement field). Note the wide variation in phase trajectories (cf. Figure 1D). **(E)** Test of the dynamic movement-field model of Equation 16 on the spike trains during all fast (red), intermediate (black), and slow (blue) gaze shifts into the movement field of the 20° unit. Compare with Figure 1.

To look at this point in a little more detail, we analyzed the static and dynamic movement-field relationships of our model SC units during eye-head gaze shifts over the full range from 1 to 60°. The results for three representative units are shown in Figure 11.

**TABLE 1:** Fitted movement-field parameters (Equation 14) for the three units shown in Figure 11B.

Cell (Δ G)	u_n_ (mm)	N_0_ (#spks)	u_0_ (mm)	σ_pop_ (mm)	ε (#spks/deg)
10	2.05	21	2.06	0.49	0.0017
20	2.85	19.83	2.85	0.47	0.0021
40	3.73	20.2	3.72	0.48	0.0053

First, we examined how much a neuron’s firing rate correlates with the instantaneous gaze velocity (cf. Figure 1C). Panel 11A shows the gaze-velocity traces (black) and instantaneous firing rates for the unit encoding *ΔG* = 20° (red) for a fixed 20° gaze shift from three initial eye positions. For illustrative reasons, the data traces were normalized with respect to their peak amplitudes and to the respective gaze durations (set at 1.0). The normalized traces appeared to correlate well, although for the ipsilateral condition the peaks of the two curves are no longer well-aligned. Overall, we obtained correlations r > 0.8 for all trials in the majority of the 200 model cells.

In Figure 11B we analyzed the static movement-field properties of three units for the three initial eye positions, by plotting the total number of spikes in the burst for each unit as a function of gaze-shift amplitude. The units were located near the rostral, central, and caudal sites of the SC, respectively. By moving from rostral to caudal areas of the map, the unit’s movement field covers a larger range of gaze-shifts, which is caused by the expansive exponential efferent neural mapping (Robinson, 1972; Ottes et al., 1986; Van Gisbergen et al., 1987; Equation 1). Thus, the 10° neuron is recruited for gaze shifts between about 6–15° amplitude (range 9°), while the 40° neuron is involved in gaze shifts between 30 and 60° (range 30°).

Note that the three units emitted a fixed number of ∼20 spikes for their optimal gaze shift and that the number of spikes varied only little (but systematically) across the different initial eye positions. For example, for the 40° neuron, with ε = 0.0053 spks/deg and E_0_ = +20° (ipsi), the expected maximum number spikes increased from 20 to 22 spikes (as 20 ⋅ 0.0053 ⋅ 20 = 2.12) and for E_0_ = −20° (contra) decreases to 18. For ipsilateral eye orientations (E_0_ > 0) the number of spikes was indeed slightly higher than for contralateral eye positions for all units. The dotted lines represent the fitted static movement-field curves through these data (Equation 14). The optimal fit parameters for the three cells are given in Table 1.

Figure 11C shows the predicted number of spikes from the static movement-field model (Equation 14) vs. the measured number of spikes for the 20° neuron (the central neuron in Figure 11B) by using its optimal parameters (Table 1; cf. Figure 1D). The static movement-field model predicts the data quite well for all three eye orientations (*r* = 0.98).

Figure 11D presents the phase plots for the spike trains for this 20° unit. It shows the cumulative number of spikes, *CS(t)*, as function of the dynamic gaze-shift vector, △*G(t+ΔT_*G*_)*, with *ΔT_*G*_* = 10 ms. Note that each trajectory has a different slope and endpoint that varied considerably for each gaze shift. Also note the influence of initial eye position on the cumulative number of spikes in the burst, which appears to organize the phase trajectories in three different clusters: blue for ipsilateral, black for central and red for contralateral initial eye positions. For saccades with enough spikes (e.g., N > 12), and for the smaller gaze shifts, the trajectories are close to linear, but for the largest saccades (△G > 24°) into the fringes of the unit’s movement field, the associated bursts ended well before the end of the gaze shifts, making their phase trajectories flatten out considerably. This latter point was typical for the units as well (see, e.g., also Figure 10C).

To quantify how the cumulative number of spikes evolves during the gaze shift, we determined the prediction of the dynamic movement-field (Equation 16) to see how well it captured the variability in the unit’s spiking behavior, observed in Figure 11D. To that end, we used the static movement-field result from Equation 14 for the total spike counts to find the slope of the dynamic phase-relation. The result for the data of panel 11D is shown in Figure 11E. Now, the prediction matches the measured trajectories quite well, as the overall correlation between the measured and predicted instantaneous spike counts is very high: *r* = 0.98 (for 591 data points).

## Discussion

We constructed and tested a simple feedback control model for horizontal primate eye-head gaze shifts that was driven in a feedforward way by the output of a spiking neural network model of the midbrain SC. In our new computational model, the initial eye-in-head orientation influenced the dynamic characteristics of the SC neurons in a uniform way, such that their bursting properties varied systematically with changes in eye position. We tuned the parameters of the neurons such that the firing rates would monotonically increase with contralateral eye positions and decrease for ipsilateral eye positions without appreciably affecting the total number of spikes in the bursts. In addition, initial eye position affected the contribution of the eye and head to the gaze shift by modulating the relative timings of the eye- and head-movement onsets in a similar (linear) way as on the SC units. Despite its simplicity, the model produced horizontal eye-head gaze shifts with realistic kinematic properties for a wide variety of initial conditions and amplitudes, together with neural response patterns in the SC motor map that faithfully resembled neurophysiological recordings from head-unrestrained monkeys.

### Superior colliculus modulations

Our earlier work had indicated that the joint tuning of three biophysical parameters of the model SC neurons in the network determine both the peak firing rate and the number of spikes in the burst: the adaptation time constant, the top-down connections from the input layer, and the strength of the lateral intracollicular interactions. For simplicity, we here let the eye-position signal only affect the intrinsic SC parameters, i.e., *τ_*q,n*_* and the excitatory/inhibitory lateral connection strengths. Quite remarkably, in tuning the network for the imposed constraints we could obtain the required neural modulations with a single, simple linear gain control on both intrinsic variables (Equations 10–12; Figure 2).

Although we did not attempt to optimally fit the neuronal firing patterns of our model units to those from real SC recordings in monkey (like in Figure 1), the overall response behaviors of our model resulted to be quite similar. For example, although the aim was to modulate only the firing rate (and burst duration), but not the number of spikes, the units nonetheless showed a small positive sensitivity on their number of spikes for eye position, since we found that the gainfield parameter ε > 0 for all neurons (e.g., Figures 5, 11B and Table 1). This led to a small increase or decrease with ΔN ∼1–2 spikes for ipsilateral (slow movements) vs. contralateral (fast movements) eye positions and for small and large gaze shifts, respectively (note that ΔN = ε⋅N_0_E_0_). Interestingly, the eye-position sensitivities of our model neurons resulted to be very similar to values obtained from real neurons in monkey. For example, for the cell in Figure 1 with *ΔG_*opt*_* = 37° we obtained ε = 0.0024 spikes/deg, and in Van Opstal and Kasap (2019) we reported ε = 0.0063 spks/deg for a cell with an optimal gaze vector of 57° amplitude.

Our model neurons yielded ε = 0.0017, 0.0021, and 0.0053 spikes/deg for the 10, 20, and 40° gaze-shift neurons, respectively. This suggests that the modest eye-position sensitivity of the number of spikes increases with gaze-shift amplitude, even though the influence of the eye-position signal on the neurons was distributed homogeneously across the motor map. The underlying mechanism for this phenomenon can be explained by the position-dependent tuning of the relevant unit-model parameters across the map as follows: The number of spikes in the burst and the peak-firing rates are determined by precise co-tuning of the adaptation time constant and lateral connection strengths (Kasap and van Opstal, 2017; Alizadeh and Van Opstal, 2022), which are both location dependent in our model. Because of this, the model produces high firing rates and short burst durations at rostral sites, vs. lower firing rates and longer burst durations at caudal sites, which underlies the non-linear main sequence of saccades (Bahill et al., 1975; Goossens and van Opstal, 2006; Goossens and van Opstal, 2012; Kasap and van Opstal, 2017; e.g., Figure 6). As a result, however, the fixed gain effect of *E*_0_ on the adaptation time constant (Equation 10) and lateral connections (Equations 11a,b) will differentially affect the bursting characteristics of these neurons too. For example, since the lateral connection gain, *S*_*n*_, is site dependent (Equation 12), the gain effect of *E*_0_ on Equations 11a,b will be stronger at rostral sites (*u*_*n*_ small) than at caudal sites (*u*_*n*_ large). Thus, the relative tuning of the two crucial unit parameters becomes slightly imbalanced for non-zero eye positions, leading to a (small) effect on the number of spikes. As this effect will be site dependent, it will therefore vary with the gaze-shift amplitude.

### Mechanisms

Although it is tempting to speculate that a similar mechanism might apply to real SC neurons, there is no evidence to support it other than that it has been shown that the number of spikes in the SC burst for ocular saccades varies with eye-in-head position (Van Opstal et al., 1995; Stuphorn et al., 2000). The neurobiological mechanism of this effect, however, remains elusive as extracellular recordings of spikes provide access to neither the intrinsic neuronal mechanisms, nor to the nature of the neuronal input. However, the simplified set of two coupled differential equations that determine the dynamics of our model neurons (Kasap and van Opstal, 2017; Alizadeh and Van Opstal, 2022) indicates that modulation of a single intrinsic parameter (the adaptation time constant) in combination with lateral feedback of spiking activity through modulated synaptic connections may suffice to produce the eye-position effects.

How such a simple mechanism could come about is unclear, but a speculative possibility could be that a presynaptic linear modulation of the eye-position signal on the SC neurons (e.g., spatially distributed shunting by an eye-position synapse on the dendrites) might influence the neuron’s membrane potential in a non-linear, multiplicative way as manifested in Equations 10 and 11a,b. It would be interesting to further explore such a possibility by modeling not only the temporal dynamics of the units (Equations 2–4), but also the spatial distribution of the different synaptic inputs. Yet, it cannot be excluded that when using the full set of Hodgkin–Huxley equations to model the neurons, modulation of other neural parameters could lead to a similar performance as the current model.

### Superior colliculus lesions

Daye et al. (2014) argued that the SC does not play a crucial role in gaze control because normometric gaze shifts can still be generated after a complete bilateral SC lesion (Schiller et al., 1980). We would like to consider the following two points: First, the alleged restoration of gaze shifts is observed after a considerable recovery period, and although qualitative inspection indicates that responses remain accurate, the kinematics are slower and reaction times prolonged. However, a precise quantification of all kinematic properties (e.g., their eye-position dependence, division of labor between eyes and head, etc.) is not available. It should also be noted that the *acute* effects of a bilateral SC lesion on saccades are dramatic. Large bilateral injections of muscimol in monkey have shown that the animal could no longer generate any visual-evoked saccades, apart from some very slow spontaneous eye movements (Hepp et al., 1993). This indicates that under normal conditions, the SC is crucial for generating rapid goal-directed saccades. This is further supported by the immediate effects of small local reversible lesions that show specific deficits in the metrics (endpoints away from the lesion) and kinematics (substantially slower) of saccades (Lee et al., 1988; Quaia et al., 1998; Goossens and van Opstal, 2006). Clearly, our model would produce similar deficits to such lesions. Second, the proposed SC mechanisms in encoding gaze shifts may be relatively straightforward to reproduce elsewhere in the brain from a neurobiological perspective: it requires learning of a simple spatial gradient of neural membrane parameters within the (alternative) motor map, in combination with a linear modulation of these parameters by eye position. It is conceivable that after a chronic and complete bilateral lesion of the colliculi another structure, e.g., the frontal eye fields, could take over with (approximately) similar neural modulations. We have not incorporated such a “back-up” system in our model.

### Gaze-shift kinematics

The stereotyped amplitude-peak velocity relationship for ocular saccades (Bahill et al., 1975) does not hold for eye-head gaze shifts (Figure 9). First, variation of initial eye position strongly affects the gaze kinematics as it is a strong determinant for the contribution of the head during the gaze shift. In the model, this is achieved by a simple linear influence of *E*_0_ on the onset delay of the head with respect to the eye. The earlier the head starts to move, the more time to influence the gaze-feedback loop by interacting with the eye-velocity signal. Thus, with the eye looking ipsilaterally to the target (*ΔT_*H*_* reduced), the head contribution is substantially larger than when looking contralaterally, causing the latter gaze shift to be faster than the former (Figure 7). Also, the planned gaze amplitude affected the head contribution by reducing the head-onset delay in proportion with ΔG (Equation 24). This latter effect, which functionally helps the system to overcome the limited oculomotor range to acquire the target, leads to the observation that the peak gaze velocity starts to *decrease* for gaze amplitudes exceeding about 40° (Figures 8, 9A). This phenomenon, which is strongest for the fastest gaze shifts (i.e., with the eye contralateral) is also clearly seen in monkey gaze-shift data (Figure 9B; Van Opstal and Kasap, 2019). Note that this effect is not caused by the modulatory effects of eye position on the SC firing rates, which still encoded a monotonically increasing non-linear desired gaze-velocity signal (Figure 6).

According to the linear ensemble-coding model for eye-only saccades, the SC firing rates of the population directly encode the instantaneous eye velocity (and hence, the stereotyped non-linear main-sequence relationship). This conclusion was based on the argument that simulating saccades with the recorded spike patterns from many neurons through Equation 1 fully explained the trajectories and instantaneous kinematics of eye saccades, even though the entire brainstem model for the oculomotor system was assumed linear. The simple spike-count model also applies to slow saccades, e.g., when SC firing rates are reduced due to changes in initial eye position (Figure 10A), for saccades to remembered targets (Peel et al., 2020), or during blinks (Goossens and van Opstal, 2006), as in all these cases the total number of spikes in the burst remained virtually invariant.

The gaze-control model presented here, however, is no longer linear although the burst generators for the eye- and head motor systems were still modeled by simple linear input-output characteristics (Figure 3). At least three non-linearities play a role in the model that potentially break with the straightforward linear transfer characteristic of the oculomotor ensemble-coding model: (i) the eye-position and gaze-amplitude dependent delay of the head-movement onset, which affects the contribution of the slower head movement to gaze shifts; (ii) the non-linear oculomotor range; (iii) the varying gain of the VOR. Yet, despite these non-linearities, the relationships between the instantaneous cumulative number of spikes of individual neurons and the ongoing planned gaze displacement remained remarkably close to linear (Figure 1D for a real recorded neuron, and Figures 10C, 11D for our model units). As a result, the dynamic gaze-movement field function adequately described the neural tuning for instantaneous gaze shifts and their kinematics for a wide variety of initial conditions (Figures 1F, 11E).

### Gaze or eye bursters?

In the model of Daye et al. (2014) the short-lead burst neurons that eventually drive the oculomotor neurons are not part of an oculomotor feedback loop but encode gaze velocity throughout. In our model, the ocular burst neurons are driven by an eye-motor error signal, just as in Robinson’s original local feedback model (Robinson, 1973; Van Gisbergen et al., 1981) and in its successors (Jürgens et al., 1981; Scudder, 1988). However, the output of our burst neurons is a *desired* velocity signal, *⋅E_*DES*_(t)*, which does not directly drive the motoneurons during gaze shifts because of the VOR. Thus, from Equation 22 one can deduce that the output of the burst generator in our model represents


(27) $${\dot{E}}_{D\,E\,S}\,\left(t\right)=\dot{E}\,\left(t\right)+g_{V}\cdot \dot{H}\,\left(t\right)=\left\{ \begin{array}{c} \dot{E}\,\left(t\right)\,\mathrm{when}\,g_{V}=0\\ \dot{G}\,\left(t\right)\,\mathrm{when}\,g_{V}=1 \end{array}\right.$$

whereas it represents a hybrid weighted velocity signal for any other value of the VOR during the gaze shift.

### Optimal control?

It has been suggested that the main-sequence relations for ocular saccades betray a speed-accuracy trade-off in combination with an undershoot strategy (Whittington et al., 1984) for the oculomotor system that optimally deals with the detrimental effects of multiplicative noise in neural control signals (Harris and Wolpert, 1998; Tanaka et al., 2006; Van Beers, 2008) and uncertainty in the peripheral visual input. By reducing the high-frequency noisy impulse from the saccadic burst generator on the eye muscles for large saccades, the system would thus avoid the danger of saccadic overshoots that would further increase the total time for the fovea to acquire the target (Harris, 1995). In our earlier work, we have argued that such a strategy would be best embedded at a level where signals are still encoded in an omnidirectional abstract vectorial format, rather than at the level of (much more complex, high-dimensional) individual muscle-control signals, and that the SC motor map could be an excellent candidate for such an optimal control principle (Goossens and van Opstal, 2012). The tight synchronization of the saccade-related bursts within the population, in combination with the apparent encoding of the saccade kinematics at the level of the motor map, seems to support this notion (Goossens and van Opstal, 2012). Indeed, simulating saccades with neural data applied to Equation 1 produced all the kinematic features and straight trajectories seen in real saccades, without having to resort to an ad-hoc saturating non-linearity and component cross-coupling schemes in the brainstem burst generators (Goossens and van Opstal, 2006).

In line with this, it stands to reason that also eye-head gaze shifts would follow an optimal control strategy, albeit that the cost function to be minimized may differ from eye-only saccades. The oculomotor system only needs to worry about speed (time to target) and accuracy (foveation), whereby energy expenditure would be of minimal importance as the eye has negligible mass. This is not true for the head, and therefore a metabolic cost (e.g., total kinetic energy expenditure) might have to be included in the total movement cost as well (Kardamakis and Moschiovakis, 2009; Saglam et al., 2011; Shadmehr and Mussa-Ivaldi, 2012).

Considering this idea, what could be the role of the eye-position signal in the SC? We here speculate that the combined modulatory influence of eye position on the SC firing rates and on the head-onset delay might be an adaptation to optimize the control costs for combined eye-head gaze shifts. As the head’s moment of inertia is considerable, and hence its initial acceleration rather slow when compared to the eye, the system aims to minimize the contribution of the head to the gaze shift to reduce metabolic costs and at the same time optimize speed. However, because of the limited eye-in-head oculomotor range, significant head movements unavoidably need to be planned for all gaze shifts exceeding about 30° when the eyes don’t look in the contralateral direction. The uncertainty (i.e., intrinsic noise) in head-movement control signals is likely to be higher than for the eye, as the latter will hardly ever be influenced by external loads or forces and has relatively simple plant mechanics with fewer muscles (only rotations). Therefore, and in line with speed-accuracy trade-off, the central command from the SC should account for the additional noise in its gaze-control signals when large head movements are needed. This would be achieved by lowering the SC firing rates (affecting speed and energy use) without (appreciably) changing the total number of spikes (which, in our model, determines gaze-shift accuracy).

## Limitations and future work

### Limitations

Although our model faithfully reproduces many of the observed characteristics of primate gaze shifts and SC neuronal activity patterns, we have not attempted to precisely fit our model to recorded data. Some discrepancies between recordings and simulations can be observed, especially in the details of the gaze-velocity profiles and unit firing rates during large and slow gaze shifts. For example, the data in Figure 1C show a high correlation between instantaneous gaze velocity and firing rate, but for ipsilateral eye orientations these profiles were often double-peaked, or with a broad shoulder. Instead, our SC firing profiles were always single-peaked (Figure 4), and this also held for our gaze shifts (Figure 11A). The latter can be explained by the relatively large oculomotor range in our simulations, which prevented the eye to run in its mechanical limits early in the gaze shift (leading to a plateau in gaze velocity). A possible reason for the former discrepancy could be that the linear modulation by initial eye position in our model may have been too simple: perhaps the eye position signal might affect the firing rates throughout the gaze trajectory, so instead of *E*_0_, one might consider *E(t)* as a modulatory signal. The firing rate might then flatten whenever the eye would run into its oculomotor limit. We have not explored this latter possibility in our simulations.

Further, the eye-head motor-control circuits in our model (Equations 17–27) were deliberately kept as simple as possible, in order not to overexplain data with an excessive number of free parameters. Many elements in the model have an influence on the details of gaze-shift kinematics: the OMR mechanism, the interaction between the eye- and head motor systems and their timings, the eye- and head motor-plant characteristics, and the precise dynamics of the VOR. We have not attempted to optimize each of these subsystems in the present study by including more elaborate data fitting or mathematical formulations.

### Future work

A further limitation of the present model is that it can only generate horizontal gaze shifts. Although computationally more costly (Kasap and van Opstal, 2018a), it is relatively straightforward to extend the SC motor map to a two-dimensional spiking neural network that enables the programming of eye-head gaze shifts in all directions.

It would be interesting to extend the downstream gaze-controllers to the full repertoire of 3D eye-head gaze shifts (i.e., horizontal, vertical, cyclo-torsional). Eye-only saccades without head movements follow the well-known kinematic constraint of Listing’s law (Tweed and Vilis, 1987), which states that all voluntary saccades are programmed as single-axis rotations whereby the axis of rotation ensures that the 3D orientation of the eye has zero cyclotorsion throughout the trajectory. We have recently developed a 3D model of the eye that closely followed Listing’s law and produced realistic main-sequence relationships and component cross-coupling for oblique saccades in all directions by applying optimal control of the physical model that minimized the total cost of speed, accuracy, and total force exerted by the six extraocular muscles on the eye during peripheral fixations (John et al., 2021).

Listing’s law, however, does not hold for head-unrestrained gaze shifts (Glenn and Vilis, 1992), not for the eye-in-head, the eye-in-space, or for the head itself. Instead, the involved motor systems are constrained by Donders’ law, which states that each 3D orientation (of eye and head and, consequently, also gaze) has a unique cyclo-torsional state, independent of the trajectory that brought it there. One reason for this is the involvement of the VOR towards the end of (and after) the gaze shift (e.g., Figures 6, 7), which requires the full 2° of freedom needed to compensate the eye movement against any change in the ongoing 3D head orientation. Behavioral experiments have demonstrated that under certain initial conditions the eye-in-head can thus even obtain a cyclo-torsional angle of about 15° during an eye-head gaze shift (Tweed et al., 1998). The underlying neural control strategies for such movements are highly nontrivial, and also require detailed knowledge of the 3D kinematics and dynamics of the eye- (John et al., 2021) and head motor plants.

Although powerful computational models have been proposed also for 3D eye-head gaze shifts (Tweed, 1997; Daemi and Crawford, 2015), so far none of these models have incorporated the putative role of the SC in 3D gaze control. Presumably, the SC motor map issues a 2D (horizontal/vertical) desired gaze-displacement vector (Van Opstal et al., 1991; Crawford and Guitton, 1997) to the brainstem—cerebellar—skeletal motor systems, from which the appropriate 3D dynamic control signals will have to be derived. Our future work will aim to extend the current 1D model of Figure 3 to a full 3D gaze-control system.

## Computer code

All code for the model simulation and data analysis routines can be obtained from the Donders Institute data repository upon reasonable request.

## Data availability statement

The original contributions presented in this study are included in the article/Supplementary material, further inquiries can be directed to the corresponding author.

## Ethics statement

This animal study was reviewed and approved by University of Rochester Animal Care and Use Committee, and fully adhered to the National Institutes of Health Guide for the Care and Use of Animals.

## Author contributions

AA prepared the figures and drafted the manuscript. AJVO conceived and designed the research. Both authors analyzed the data, interpreted the results of the experiments, edited and revised the manuscript, and approved the final version of manuscript.
