# Supplementary material for: Dynamic control of eye-head gaze shifts by a spiking neural network model of the superior colliculus
Source: Front Comput Neurosci. 2022 Nov 17;16:1040646. doi: 10.3389/fncom.2022.1040646 (PMC9714624; doi:10.3389/fncom.2022.1040646)
Supplement: Supplementary file 1 [file Data_Sheet_1.docx]

# Supporting Information for:

**Dynamic Control of Eye-Head Gaze Shifts by a Spiking Neural Network Model of the Superior Colliculus**

Arezoo Alizadeh and A. John Van Opstal

Donders Centre for Neuroscience

Radboud University

Heyendaalseweg 135, HG00.134

6525 AJ Nijmegen

The Netherlands

In this Supplemental Materials section, we provide some additional information on the experimental paradigm for the monkeys. We briefly describe how we fitted the static and dynamic movement field of recorded SC neurons. Finally, we provide the details of the model parameter settings of the units in the input layer and SC motor map. More detailed background information can be obtained from our earlier work (Kasap and Van Opstal, 2017).

**1. Monkey gaze-control paradigm**

To vary the movement kinematics of gaze shifts, monkeys elicited gaze-saccades from different initial eye-in-head orientations. At the start of a trial, the animal directed the eyes at a straight-ahead fixation LED at 1.0 m from the animal’s head, while aligning one of three head-fixed lasers with the fixation point (Fig. S1). The lasers were positioned such that the horizontal head orientation with respect to straight ahead (FIX) would be either [-18, 0, +18] deg. For example, a target presented at 60 deg leftward, resulted in three different 60 deg eye-head gaze shifts: the head at +18 deg (i.e., laser 2, with the eyes directed -18 deg ipsilateral to the target), 0 deg (laser 1; eye-head alignment), or -18 deg (laser 3; the ‘eye-contra’ condition, +18 deg).


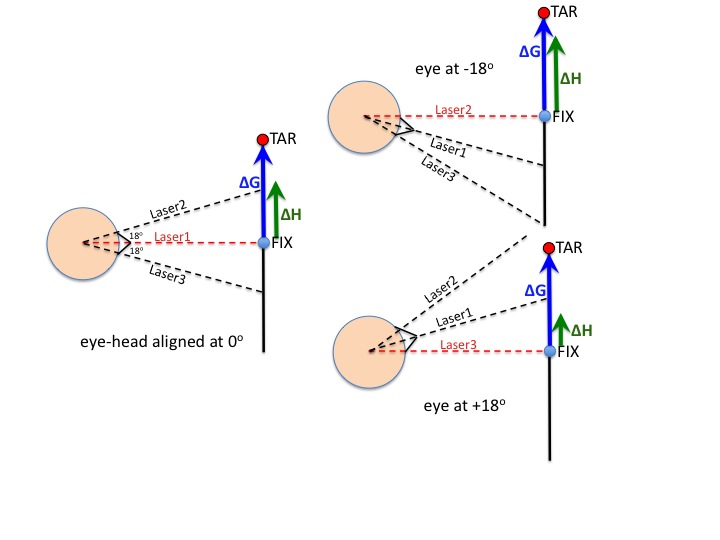
**Figure S1**. *Schematic of the paradigm, illustrating the use of the three head-fixed lasers to manipulate the initial eye-in-head orientation for an upcoming (here leftward) gaze saccade, ∆G, which is the same for all three conditions. The eyes initially look at FIX in each condition. With laser 1 also pointing at FIX, eyes and head are aligned (left). ∆H is the head movement during the gaze shift. When laser 2 points at FIX, the eyes are directed ipsilaterally to the target (-18^o^), which will elicit a larger head movement (and slower gaze shift; upper-right). When Laser 3 points to FIX, the eye-in-head is directed contralaterally from the target, leading to a smaller head movement (and faster gaze shift; lower right).*

***2.* Neural Data Analysis.**

To determine the optimal movement field parameters of a recorded neuron, gaze saccades were elicited in and around the cell’s movement field (see, e.g., Fig. 1B). We counted the number of spikes in the burst from 20 ms before gaze-shift onset to 20 ms before offset (e.g., Fig. 1A), and applied the afferent mapping function of (Ottes and Van Gisbergen, 1986) to each gaze shift to calculate its anatomical coordinates, *(u,v)* in mm, in the SC map. In polar coordinates (ΔG, Φ), this mapping reads:

$$u=B_{u}\cdot\ln\left( \frac{\sqrt{{\Delta G}^{2}+2A\cdot\Delta G\cdot cos\Phi+A^{2}}}{A} \right) \text{mm}$$

(S1)

$$v=B_{v}\cdot\mathrm{atan} \left( \frac{\Delta G\cdot\sin\Phi}{\Delta G\cdot\cos\Phi+A} \right) \text{mm}$$

where *B_u_* =1.4 mm, *B_v_* =1.8 mm/rad, and *A* = 3.0 deg determine the shape of the monkey afferent mapping function (Robinson, 1972; Ottes and Van Gisbergen, 1986).

We first fitted the *static movement field* function to all recorded gaze-saccade vectors (see Fig. 1E), and included a potential eye-in-head gain-field modulation (Van Opstal et al., 1995) by the initial eye position, E_0_, to the total number of spikes in the burst, *N*, according to:

$N\left( \Delta G,\Phi,E_{0} \right)=N_{0}\cdot(1+\varepsilon\cdot E_{0})\cdot\exp\left( -\frac{{(u-u_{0})}^{2}+{(v-v_{0})}^{2}}{2\sigma_{P}^{2}} \right)$ (S2)

This model has five free parameters: *N_0_* is the number of spikes in the burst for the optimal saccade from straight ahead, (*u_0_,v_0_*) (in mm) are the SC coordinates of the optimal saccade (Eqn. S2), *ε* (in #spikes/deg) is the eye-position gain, and *σ_P_* (in mm) quantifies the cell’s tuning width. Optimal parameter values were obtained with the Nelder-Mead Simplex algorithm in Matlab.

Next, the *dynamic* movement field describes how the cumulative number of spikes in the burst evolves during the straight gaze-displacement along the line connecting start- and end-positions (Goossens and Van Opstal, 2006). According to this model, the cumulative spike count for any gaze shift, regardless its kinematics, obeys the following, linear, relation:

$CS\left( \Delta G,\Phi,E_{0},t \right)=\Delta G(t+\tau)\cdot\frac{N(\Delta G,\Phi,E_{0})}{\Delta G}$ (S3)

where $\Delta G(t+\tau)$ is the desired straight trajectory (increasing monotonically from 0 to *ΔG*). The neuron’s lead time, $\tau$, was fixed at $\tau$ = 20 ms for all neurons. The straight trajectory was obtained by projecting the actual trajectory $\left( x\left( t \right), y\left( t \right) \right)$onto gaze vector $\Delta G\cdot\left( \cos\Phi, \sin\Phi\right)$ (Goossens and Van Opstal, 2006):

$$\Delta G\left( t \right)=x(t)\cdot\cos\Phi+y\left( t \right)\cdot\sin\Phi\text{(S4)}$$

The time-independent factor in Eqn. S3, $N(\Delta G,\Phi,E_{0})/\Delta G$, corresponds to the slope of the dynamic phase-relation (Fig. 1D). It is expected to vary in a systematic way with gaze-shift amplitude and direction (Fig. 1D,F; Goossens and Van Opstal, 2006).

***3.* Model parameters of the Spiking Neural Network**

**Table** **S1** Overview of all parameters used in the spiking network simulations

| **Input current** |  |  |
| --- | --- | --- |
| σ_p_ | 0.05 mm | Recruited input population size |
| β | 0.03 s^-1^ | Measure for input duration |
| γ | 1.8 | Skewness of input current profile |
| I_0_ | 3 pA | Input stimulation strength |
| **Input neuron parameters** |  |  |
| C | 50 pF | Membrane capacitance |
| g_L_ | 2 nS | Leak conductance |
| E_L_ | -70 mV | Leak reversal potential |
| V_T_ | -50 mV | Spike initiation threshold |
| Vpeak | -30 mV | Practical spiking threshold |
| η | 2 mV | Spike slope factor |
| *a* | 0 nS | Subthreshold adaptation |
| *b* | 60 pA | Spike-triggered adaptation |
| V_r_ | -55 mV | Resting potential |
| τ_q_ | 30 ms | Adaptation time constant |
| **SC neuron parameters** |  |  |
| C | 280 pF | Membrane capacitance |
| g_L_ | 10 nS | Leak conductance |
| E_L_ | -70 mV | Leak reversal potential |
| V_T_ | -50 mV | Spike initiation threshold |
| Vpeak | -30 mV | Practical spiking threshold |
| η | 2 mV | Spike slope factor |
| a | 4 nS | Subthreshold adaptation |
| b | 80 pA | Spike-triggered adaptation |
| V_r_ | -45 mV | Resting potential |
| τ_q_ | 1-60 ms | Adaptation time constant |
| **SC synapse parameters** |  |  |
| E_e_ | 0 mV | Excitatory reversal potential |
| E_i_ | -80 mV | Inhibitory reversal potential |
| τ_e_ | 5 ms | Excitatory conductance decay |
| τ_i_ | 10 ms | Inhibition conductance decay |
| w_n_^FS^ | 4-10 nS | Feedforward synaptic strengths (varies) |
| **Mexican hat parameters** |  |  |
| $w^{exc}$ | 0.16 nS | Excitatory scaling factor |
| $w^{inh}$ | 1.15 nS | Inhibitory scaling factor |
| σ_exc_ | 0.2 mm | Range of excitatory synapses |
| σ_inh_ | 0.7 mm | Range of inhibitory synapses |

**References:**

Goossens H, Van Opstal AJ. (2006). Dynamic ensemble coding of saccades in the monkey superior colliculus. *J Neurophysiol.* 95(4):2326-2341.

Kasap B, Van Opstal AJ. (2017). A spiking neural network model of the midbrain superior colliculus that generates saccadic motor commands. *Biol Cybernet.* 111, 249-268, 2017.

Ottes FP, Van Gisbergen JAM. (1986). Eggermont JJ. Visuomotor fields of the superior colliculus: a quantitative model. *Vision Res.* 26(6):857-873

Robinson DA. (1972). Eye movements evoked by collicular stimulation in the alert monkey.

*Vision Res.* 12(11):1795-1808

Van Opstal AJ, Hepp K, Suzuki Y, Henn V. (1995). Influence of eye position on activity in

monkey superior colliculus. *J Neurophysiol.* 74: 1593-1610
